# Correlates of degree of nerve involvement in early Bell's palsy

**DOI:** 10.1186/1471-2377-9-22

**Published:** 2009-06-07

**Authors:** Ru-Lan Hsieh, Chia-Wei Wu, Ling-Yi Wang, Wen-Chung Lee

**Affiliations:** 1 Department of Physical Medicine and Rehabilitation, Shin Kong Wu Ho-Su Memorial Hospital, Taipei, Taiwan; 2 Taipei Medical University, Taipei, Taiwan; 3 Graduate Institute of Epidemiology, College of Public Health, National Taiwan University, Taipei, Taiwan

## Abstract

**Background:**

This study aimed to evaluate the still unknown factors correlating with the degree of nerve involvement in early Bell's palsy.

**Methods:**

This retrospective chart review study of newly diagnosed cases of Bell's palsy was conducted over a three-year period. Information on age, sex, day of onset, comorbidities, corticosteroid use, and electroneurographic test results were collected. The electroneurographic quotient (amplitude of compound muscle action potential on the affected side divided by that on the healthy side and expressed in percent) was used as an index of nerve involvement, with lower quotient indicating more severe disease.

**Results:**

Data were collected on 563 patients. The mean electroneurographic quotient varied inversely with age (*p *< 0.001) and was higher in patients who used corticosteroids than those who did not (47.1% *vs*. 40.3%; *p *= 0.002). There was no correlation between the degree of nerve involvement and sex, season of onset, hypertension, or diabetes.

**Conclusion:**

The degree of nerve involvement in early Bell's palsy correlates positively with age and negatively with corticosteroid use.

## Background

A concern of both patients with Bell's palsy and their physicians is when and how facial paralysis will be completely resolved [1]. Many previous studies have focused on the long-term prognosis of Bell's palsy [1-8]. Systems for clinically assessing prognosis include the House-Brackmann facial nerve grading system, Yanagihara scoring system, nerve excitability test, electroneurography, electromyography, blink reflex, stapedial muscle reflex, prediction equations using combinations of these, brain magnetic resonance imaging, and even a novel 3-dimensional real-time video acquisition system [1-3,7-12]. Of these, electroneurography is the most frequently used because it provides objective, quantitative, and accurate data for assessing facial nerve function [1,6]. It measures and records the amplitudes of muscle summation potentials resulting from the synchronous firing of motor units [6]. A percent of nerve fibers that are neuropraxic is obtained by comparing the amplitude of the compound muscle action potentials on the affected side with that on the healthy side [8,13].

Electroneurography has been used in many clinical studies to evaluate compound action potentials, nerve conduction velocities, and distal motor conduction latencies on two sides of the face [14-16] and thereby to detect early-stage conditions involving neural damage. The purpose of these studies was mainly to predict long-term follow-up prognosis and select subjects for treatment [17]. Long-term prognostic studies of Bell's palsy have shown that recovery rate in older individuals is poor [1,5,18]. Unlike the study of Salinas et al [19], many studies show usage of corticosteroids improves the prognosis of Bell's palsy [4,10,16,20-22]. Nearly everyone agrees that mild early Bell's palsy carries a better long-term prognosis. However, indicators of the degree of nerve involvement in early Bell's palsy are unknown. Therefore, our aim was to evaluate potential factors (age, sex, hypertension, diabetes mellitus, season of onset, and corticosteroid use) as correlates of the degree of nerve involvement in early Bell's palsy.

## Methods

This retrospective chart review study was of cases of Bell's palsy newly diagnosed between 2003 and 2005 at Shin Kong Wu Ho-Su Memorial Hospital (a teaching hospital in Taipei, Taiwan). The Bell's palsy diagnostic criteria were acute onset of lower motor neuron facial palsy unaccompanied by evidence of traumatic, vascular, oncologic, or other infectious etiologies ruled out by aural, neurologic, or clinical examination [23]. The research ethics committee of the hospital approved the study protocol. Data on age, sex, day of onset, comorbidities (e.g., hypertension, diabetes mellitus, hepatic disease, renal disease, and cardiovascular disease), corticosteroid use, and treatment with acyclovir post-diagnosis were collected. Electroneurographic studies were performed 10–15 days after disease onset using a 2-channel Medelec™ Synergy N-EP – EMG/EP Monitoring System (Oxford Instruments Medical, Oxford, UK). Facial nerves were stimulated bilaterally with bipolar surface electrodes placed over the stylomastoid foramen and their responses were recorded from electrodes placed over the orbicularis oculi muscles. Maximal compound muscle action potentials were obtained by gradually increasing the intensity of the stimulus to supramaximal levels [13]. An electroneurographic quotient, defined as the ratio of the amplitude of compound muscle action potential on the affected side to that on the healthy side [7], was calculated (as a percent) and served as the index of degree of nerve involvement. A lower electroneurographic quotient indicates more severe disease.

The chi-square test was used for statistical analysis of categorical variables and Student's *t *test or analysis of variance was used for continuous variables. A difference was considered significant at a *p*-value of less than 0.05. Correlates of electroneurographic quotient were analyzed by multiple regression analysis.

## Results

A total of 787 cases of Bell's palsy were reviewed. Twelve cases of recurrent Bell's palsy and 212 cases without electroneurographic test data were excluded. Data from a total of 563 cases (319 men; 244 women) were included. The age distribution in years was 0–90 with 33 patients (aged 0–20), 102 (aged 21–30), 104 (aged 31–40), 105 (aged 41–50), 98 (aged 51–60), 73 (aged 61–70), and 48 (71–90). The season of disease onset was spring (for 115 patients), summer (114), autumn (145), and winter (159). A total of 61 patients (10.8%) had hypertension, 38 (6.7%) had diabetes mellitus, and 24 (4.3%) had both. Corticosteroids were used by 347 (61.6%) patients and 11 patients used both corticosteroids and antiviral drugs at the same time. The demographics were similar between excluded patients without electroneurographic data and included patients with electroneurographic data. More women than men underwent electroneurographic studies.

**Figure 1:**
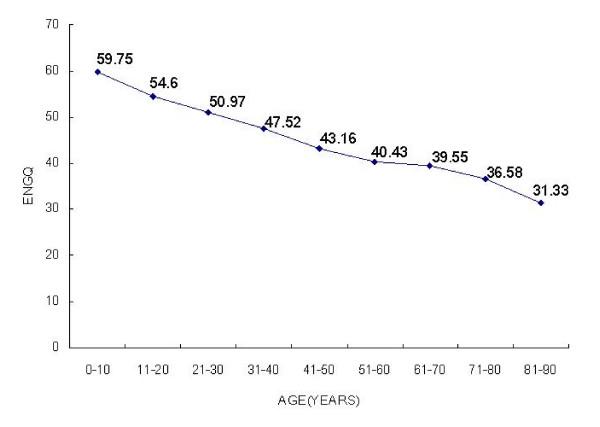
**The correlation of electroneurographic quotient (ENGQ) with age**. Electroneurographic quotient decreased significantly with increase in age (*p *< 0.001).

The correlation of electroneurographic quotient with study variables is shown in Table 1. Electroneurographic quotients decreased significantly with increase in age (*p *< 0.001), indicating more severe disease occurred in older patients (Table 1, Figure 1). Patients who used corticosteroids had better (i.e., higher) electroneurographic quotients than those who did not (47.1% *vs*. 40.3%, *p *= 0.002). Electroneurographic quotient was not correlated with sex, season of onset, hypertension, or diabetes mellitus. Multiple regression analysis showed a decrease in electroneurographic quotient of 2.78 (*p *< 0.001) per 10-year increase in age, and an increase in electroneurographic quotient of 5.47 (*p *= 0.010) in patients treated with corticosteroids.

**Table 1:** The correlation of electroneurographic quotient with clinical and demographic features in 563 subjects with Bell's palsy.

Variables	Electroneurographic quotient mean(SD)	*p *value
Sex		
Men	44.3 (24.6)	0.77
Women	44.9 (24.9)	
Age (in years)		
0–10	59.8 (15.0)	<0.001**
11–20	54.6 (26.3)	
21–30	60.0 (23.8)	
31–40	47.5 (24.8)	
41–50	43.2 (24.6)	
51–60	40.4 (21.9)	
61–70	39.5 (26.9)	
71–80	36.6 (23.5)	
81–90	31.3 (25.1)	
Onset season		
Spring	46.1 (24.5)	0.676
Summer	55.0 (24.0)	
Autumn	45.7 (24.4)	
Winter	43.1 (26.0)	
Hypertension		
yes	50.4 (26.2)	0.05
no	43.8 (24.5)	
Diabetes mellitus		
yes	45.3 (27.7)	0.835
no	44.5 (24.5)	
Corticosteroid use		
yes	47.1 (24.2)	0.002*
no	40.3 (25.1)	

*p < 0.005, **p < 0.001

## Discussion

Following complete transection of the facial nerve, Wallerian degeneration starts in 3–5 days and maximal injury usually occurs within 1–2 weeks [3,7,8,24]. For this reason, most clinical neurophysiologists recommend carrying out electromyographic and electroneurographic studies 10–15 days after the onset of facial weakness in Bell's palsy to determine the magnitude of axonal damage to the facial nerves [25]. Since this time window (10–15 days after onset of Bell's palsy, which is coincident with axonal degeneration in early Bell's palsy) was routinely used in our hospital, we were able to obtain the data needed to examine the correlation of electroneurographic quotient with possible prognostic factors.

Older patients with Bell's palsy have poor prognoses [1,5,18]. Our study showed that the degree of nerve involvement in early Bell's palsy increased with increasing age. Less severe nerve damage in younger patients could also be due to early use of corticosteroids. To avoid this bias, patients were stratified according to corticosteroids use for further analysis. The correlation of electroneurographic quotient with age was -0.223 (*p *< 0.001) in 347 patients treated with corticosteroids, and -0.159 (*p *= 0.019) in 216 patients without corticosteroids therapy, indicating that the degree of nerve involvement was negatively correlated with age, irrespective of corticosteroids use.

Aging diminishes the capacity for neural regeneration [5]. This could be due to glial cell hyperactivity and an aging-related increase in brain cytokine activity that impairs the ability of cells to repair themselves [26]. Animal studies show that aging is associated with increased reactivity of astrocytes and decreased post-lesion sprouting capability [27]. In addition, the basal level of intracellular calcium in older animals is increased, which decreases neural network efficiency [28]. All of these factors could contribute to the more severe facial nerve damage in older patients [29].

Our study showed that use of corticosteroids decreased the degree of nerve involvement in early Bell's palsy. While the dosages and durations varied, corticosteroid therapy was started within 3 days of onset in most cases. Although some older studies report equivocal findings, more recent studies show that early corticosteroid treatment of Bell's palsy (within 3 days, and up to 7 days from onset) improves long-term recovery [16,22,30,31]. Although electroneurographic studies performed 10–15 days after onset could be affected by differences in corticosteroid dosage and duration, on average, electroneurographic quotient was 7% higher in patients who used corticosteroids.

In this study, 61.6% of patients received corticosteroid therapy. The corticosteroid-treated group had more men (*p *= 0.030) than women and were younger (*p *= 0.040) than the group that did not receive corticosteroids. Since gender had no effect on the relationship of electroneurographic quotient to degree of nerve involvement, the presence of a greater number of corticosteroid-treated male patients did not confound our analysis. Nonetheless, this retrospective study could not avoid bias that could arise from the possible preferred use of corticosteroids in younger and healthier patients because corticosteroids have fewer contraindications in younger and healthier patients. On the other hand, patients might be given corticosteroids because they were more severely affected. Then, the observed difference in electroneurographic quotients in this study may underestimate the effect of corticosteroid therapy. Also, because this is not a randomized study, it remains unclear whether the higher electroneurographic quotient detected in the corticosteroid group is attributable to spontaneous remission or to actual therapeutic effects of corticosteroids.

Although Merwarth [32] suggested that hypertension increased the incidence of bleeding within the fallopian canal and facial nerve compression, the relationship between hypertension and the severity of Bell's palsy remains controversial [5]. We found borderline significant correlation between the electroneurographic quotient and hypertension (*p *= 0.050). Further, hypertension-stratified analysis of electroneurographic quotient showed that corticosteroid-treated subjects with hypertension had significantly better electroneurographic quotients than corticosteroid-treated subjects without hypertension (54.9% *vs*. 46.2%, *p *= 0.044). Electroneurographic quotient was negatively correlated with age (correlation coefficient, -0.0065; *p *< 0.0001) in non-hypertensive patients. Electroneurographic quotient was not correlated with gender in our hypertensive group. Nonetheless, the number of subjects with hypertension in our study (n = 61) was small, and therefore generalization to larger patient populations might be unwarranted. Our results were similar to those of a previous study reporting no definitive correlation between diabetes mellitus and severity of Bell's palsy [5].

Fisch et al. found that when the electroneurographic quotient was more than 10% (i.e., the extent of facial nerve degeneration was less than 90%), all patients recovered fully; when it was 5%, half of patients had a poor recovery on long-term follow up [2,3,6,7]. In contrast, May and Chow found that if the electroneurographic quotient was 27–30%, patients had an 84–90% chance of a good recovery [8,33]. Although long-term prognostic results of electroneurographic quotient differed due to variations in the studied populations [24], the electroneurographic quotient correlated well with the degree of nerve involvement in early Bell's palsy [1,6,8].

In our study, electroneurographic quotient was used as an index of nerve involvement in early Bell's palsy. The facial nerve contains about 10,000 fibers [3]. The amplitude of compound muscle action potential is measured peak-to-peak in micro volts [3]. Many factors such as electrode placement, electrode application pressure, skin resistance, and stimulating current affect electroneurographic study results [1,34]. Variation in electroneurography of 3–5.4% between the two sides of the face (i.e., right side *vs*. left side) and 10% between tests [35,36] has been reported. In our hospital, all electroneurographic studies were performed in two electrodiagnostic laboratories by skilled technicians in the Department of Neurology and by physiatrists in the Department of Physical Medicine and Rehabilitation. Therefore, we believe that the possible small variations in electroneurographic studies have not adversely affected our findings.

This retrospective study had some limitations, such as differences between subgroups, small size of some subgroup samples, and bias of administering corticosteroids mainly to younger subjects. In addition, this study lacks measurements of long-term clinic outcome. In the 563 subjects, the average number of clinic visits was 3.5 (ranging from only one visit in 13 subjects to a maximum of 27 visits), with a mean of 53.3 days (ranging from 0 days to 2.9 years) of follow up. These subjects mainly visited departments of neurology, otolaryngology, physical medicine and rehabilitation, and some visited departments of family medicine and internal medicine. Although nearly all subjects showed fair to good recovery by clinical physical and neurological examination recorded by the diagnosing and treating physicians, no uniform objective clinical measurements were used. Therefore, our data can not be used to identify correlates with long-term prognosis.

## Conclusion

We assessed the factors correlating with the degree of nerve involvement in early Bell's palsy. We found a positive correlation with age and negative correlation with corticosteroids use. However, this was a retrospective study. To evaluate the relationship of corticosteroid therapy to the degree of nerve involvement in early Bell's palsy, future prospectively randomized studies of corticosteroid therapy with an appropriate method (e.g., clinical scores) are essential.

## Competing interests

The authors declare that they have no competing interests.

## Authors' contributions

RLH carried out the design of the study and drafted the manuscript. CWW participated in the design of study and data collection. LYW participated in the design of the study and performed the statistical analysis. WCL participated in the study of design and coordination. All authors read and approved the final manuscript.

## Pre-publication history

The pre-publication history for this paper can be accessed here:


